# Editorial Recreations

**Published:** 1856-06

**Authors:** 


### EDITORIAL RECREATIONS.

SIR:—Three months ago I sent you a year's subscription, as I can prove by the Post-master of this town; not a single number has been received. As I don't want to be trifled with, I hope your publication will be sent at once to

MISS MARY FLANDERS.

*Reply.*—MISS MARY:—The Journal has been regularly sent to your address at *Mary-Gold Cottage*, such being the designation of your first note. We send them again, however, to the Post-office, the official impress of which is on the envelope of your last.

*Journal Editor:—*

Sir:—I have only received the January number, and I know by that that you have pocketed the dollar, and as I don't choose to pay a whole dollar for one number of your Journal, this is to express the hope that you will be honest and send them regularly.

Jemima Peppercorn.

*Reply.*—The name of your Post-office having been changed without our having been made acquainted with the fact, the Journal was sent to your old address. We however mail the missing numbers as per present letter.

*Editor of Journal of Health:—*

I have been at the trouble to obtain a number of subscribers for you. I have so many complaints about the tardy or non-reception of the Journal, that I don't think I will be troubled with it any more.

Miss Powderflask.

*Reply.*—My Dear Miss Powdry:—Your appreciated communication is duly received, and in reply thereto beg leave to say that we agree to mail the Journal regularly to all of our subscribers, but we do not undertake to transport the United States mail. Your difficulty is with Uncle Sam; all that we can do in such cases, and we shall do it promptly and willingly, is to supply missing numbers to all of our subscribers, without additional charge, on being made acquainted with the fact that any number has not been received.

*Dr. Hall:—*

Your Journal comes as an exchange to the office of this Magazine, in Chestnut-street, Philadelphia, and I am much pleased with it, but it is so cut up for copy I desire the bound volume, the price of which is enclosed. Will you please to tell me what will fetch down a big head; mother says mine is a great deal larger than it ought to be, and that it was caused by eating so much molasses and candy and sweet cake, and I will be very much obliged to you. I am nineteen just.

Richard Countryman.

*Reply.*—Master Richard:—The Journal is sent as desired. I know of no means of diminishing the size of your head, but

I can suggest a sufficient remedy; fill the head you have with substantial knowledge, and by a life of active industry out of doors, eating heartily of substantial meat and bread and fruits, make your body grow up to it—then the relative disproportion in size between the head and body will not be so great as to attract notice.

Very truly yours,
ED. HALL'S J. HEALTH.

*Editor Journal of Health:—*

I have seen so many extracts from your Journal, will you please send me five or six different numbers as a specimen and if I like it, and can spare the money, and could be sure that some one would not take it out of the letter, I think I might like to subscribe for it next year, if father will let me have the money.

JOHN KEEN.

We mailed John Keen a last year's Journal so that if he liked it, &c., his promise to take it "*next*" year would make him a subscriber to the present number.

*Dr. Hall:—*

I like your Journal very much and would like to take it, but cannot spare the money just now; but if you will send it to me, I will send you the money next fall, when the crops come in. WILLIAM SUREMAN.

The above was received in the summer of last year, from the interior of Ohio, and the Journal was sent accordingly. When the crops were gathered the dollar was sent. And we record this as a striking instance of the looming up of a dollar, in the estimation of people in the country who do not handle much money.

